# Genetic variation in the ATP binding cassette transporter *ABCC10* is associated with neutropenia for docetaxel in Japanese lung cancer patients cohort

**DOI:** 10.1186/s12885-019-5438-2

**Published:** 2019-03-19

**Authors:** Kazuki Sone, Tetsuya Oguri, Takehiro Uemura, Akira Takeuchi, Satoshi Fukuda, Osamu Takakuwa, Ken Maeno, Kensuke Fukumitsu, Yoshihiro Kanemitsu, Hirotsugu Ohkubo, Masaya Takemura, Yutaka Ito, Akio Niimi

**Affiliations:** 1 Department of Respiratory Medicine, Allergy and Clinical Immunology, Nagoya City University Graduate School of Medical Sciences, 1 Kawasumi, Mizuho-cho, Mizuho-ku, Nagoya, 467-8601 Japan; 2 Department of Education and Research Center for Community Medicine, Nagoya City University Graduate School of Medical Sciences, 1 Kawasumi, Mizuho-cho, Mizuho-ku, Nagoya, Aichi 467-8601 Japan

**Keywords:** ABC transporter, Single nucleotide polymorphism, Docetaxel, Neutropenia

## Abstract

**Background:**

Docetaxel is a widely used cytotoxic agent for treatments of various cancers. The ATP binding cassette (ABC) transporter / multidrug resistance protein (MRP) ABCC10/MRP7, involved in transporting taxanes, has been associated with resistance to these agents. Since genetic variation in drug transporters may affect clinical outcomes, we examined whether polymorphism of *ABCC10* could affect clinical responses to docetaxel.

**Methods:**

Using 18 NSCLC cell lines and CRISPR-based genome-edited HeLa cells, we analyzed whether genetic variants of *ABCC10* (rs2125739, rs9349256) affected cytotoxicity to docetaxel. Subsequently, we analyzed genetic variants [*ABCC10* (rs2125739), *ABCB1* (C1236T, C3435T, G2677 T/A), *ABCC2* (rs12762549), and *SLCO1B3* (rs11045585)] in 69 blood samples of NSCLC patients treated with docetaxel monotherapy. Clinical outcomes were evaluated between genotype groups.

**Results:**

In the cell lines, only one genetic variant (rs2125739) was significantly associated with docetaxel cytotoxicity, and this was confirmed in the genome-edited cell line. In the 69 NSCLC patients, there were no significant differences related to rs2125739 genotype in terms of RR, PFS, or OS. However, this SNP was associated with grade 3/4 neutropenia (T/C group 60% vs. T/T group 87%; *P* = 0.028). Furthermore, no patient with a T/C genotype experienced febrile neutropenia.

**Conclusions:**

Our results indicate that genetic variation in the *ABCC10* gene is associated with neutropenia for docetaxel treatment.

## Background

Docetaxel, an inhibitor of microtubule depolymerization, has shown significant efficacy in various cancers. For example, clinical trials in patients with non-small cell lung cancer (NSCLC) have shown that docetaxel is active not only in front-line chemotherapy or chemoradiotherapy combined with platinum drugs [1–3], but also in previously treated patients [4]. The safety profile of docetaxel is well-defined, with its main dose-limiting toxicities being neutropenia and/or neutropenic fever [5, 6]. In addition, it seems that lower doses of docetaxel are less active than higher ones [7].

Drug transporters, such as ATP binding cassette (ABC) transporters, occupy an important place in drug metabolism. These transmembrane proteins are involved in the transport of biologically important substrates, including anti-cancer agents [8], across cell membranes, and they can therefore affect the treatment outcome of chemotherapy. For example, overexpression of ABC transporters can increase drug efflux and decrease cytoplasmic drug concentration, which results in reduced efficacy and potentially a drug resistant phenotype. Furthermore, drug transporters influence anti-cancer pharmacokinetics, resulting in chemotherapy-induced adverse effects, such as neutropenia [9]. It has been reported that the influx transporter SLCO1B3 and the efflux transporters ABCB1 and ABCC2 have important roles in docetaxel metabolism [10]. Moreover, there are several reports that single nucleotide polymorphisms (SNPs) in these transporter genes influence the clinical outcome of patients treated with this agent [11–13]. On the other hand, we have previously reported that ABCC10/multidrug resistance protein 7 (MRP7) can confer resistance to anti-tubulin agents, including taxanes [14–16]. ABCC10/MRP7 is an efflux transporter highly expressed in liver, intestine, and peripheral blood cells [17], indicating that it plays a physiological role in normal tissue detoxification. *ABCC10* has genetic variants that have been shown to contribute to variability in the plasma concentration of anti-viral drugs or response to oxaliplatin in colorectal cancer [18, 19]. However, it is not yet known whether *ABCC10* polymorphisms might affect clinical outcome following treatment with docetaxel. In this study, we investigated whether genetic variation in *ABCC10*, compared with the SNPs of other docetaxel transporter genes, contributes to the efficacy and/or safety of docetaxel treatment.

## Methods

### Cell lines and chemicals

The following human NSCLC cell lines were used in this study: 13 adenocarcinoma lines (A549, NCI-H23, PC-9, PC-14, VMRC-LCF, RERF-LC-AI, RERF-LC-MT, RERF-LC-OK, RERF-LC-MS, NCU-LC-201, ACC-LC-94, ACC-LC-176, ACC-LC-314), three squamous cell carcinoma lines (PC10, QG56 and Calu1), and two large-cell carcinoma lines (NCI-H460 and SK-LC-6). These cell lines and HeLa cells were provided by Aichi Cancer Center. Cells were cultured in RPMI 1640 supplemented with 10% heat-inactivated FBS and 1% (*v*/*w*) penicillin/streptomycin in a humidified chamber (37 °C, 5% CO2). Docetaxel and verapamil were purchased from Wako Pure Chemical Industries (Osaka, Japan).

### Drug sensitivity assays

NSCLC cells were diluted 5000 cells/100 μl and were seeded in 96-well tissue culture plates. Then, stepwise ten-fold dilutions of docetaxel were added 2 h after plating, and the cultures were incubated at 37 °C for 72 h. Cell survival rates were determined using MTS [3-(4,5-dimethylthiazol-2-yl)-5-(3-carboxymethoxyphenyl)- 2-(4-sulfophenyl)-2H-tetrazolium, inner salt] solution assay (CellTiter 96® AQueous One Solution Cell Proliferation Assay, Promega, Madison, WI). The absorbance was measured at 490 nm using an ELISA plate reader. These assays were performed as described previously [20]. Mean values were calculated from three independent experiments carried out in triplicate. Chemosensitivity is expressed as the IC_50_ (drug concentration resulting in 50% growth inhibition), determined using Graph Pad Prism version 4 (GraphPad Software, San Diego, CA).

### CRISPR-Cas9 genome editing

To change the *ABCC10* rs2125739 SNP from wild type to variant in HeLa cells with the wild type sequence at that location, we purchased custom-designed gRNA (target sequence gRNA1; ACGGATGTCTGAGGAGCCAT, gRNA2; GATGTCTGAGGAGCCATTGG) from ThermoFisher Scientific Life Technologies Japan (Tokyo, Japan). Specifically, HeLa cells were co-transfected with Cas9 protein (Invitrogen, Carlsbad, CA) and Donor DNA (with the “C” allele SNP sequence) according to the instructions included with lipofectamine CRISPRMAX (Invitrogen). After 48 h, the cells were transferred to a 96-well culture plate for clonal selection. DNA was isolated from the transfected cells and the DNA sequence of each clone carrying the “C” allele was determined by Sanger sequencing. This procedure allowed us to obtain a HeLa CRISPR1 (T/C) cell line (using gRNA1) that was heterozygous for the *ABCC10* rs2125739 SNP genotype and a HeLa CRISPR2 (C/C) cell line (using gRNA2) that was homozygous minor allele genotype within the SNP. Docetaxel sensitivity was measured in the HeLa parent cells and HeLa CRISPR-edited cells in the presence of 20 μM verapamil, added to the medium as described previously [20]. We carried out five or less subcultures until we established CRISPR-edited cells from HeLa parent cells.

### Western blotting

Cells were lysed in sample buffer (50 mM Tris-HCl (pH 6.8), 2% SDS, 1 mM EDTA, and 10% glycerol) with Complete Mini Protease Inhibitor Cocktail Tablets (Roche Diagnostics, Mannheim, Germany) and PhosSTOP Phosphatase Inhibitor Cocktail Tablets (Roche Diagnostics). Subsequently, equal amounts of protein were applied to 7.5% Ready Gel Tris-HCl Precast Gels (Bio-Rad Laboratories, Hercules, CA) and electrophoresed. Then, that transferred onto Immobilon-P filters (Millipore, Billerica, MA). The filters were first incubated with primary antibodies against ABCC10/MRP7 (160 kDa) and α-tubulin (50 kDa) overnight at room temperature and then with horseradish peroxidase (HRP)-conjugated secondary antibodies for 1 h. Chemiluminescence images were captured on ImageQuant LAS4000 (Fujifilm, Tokyo, Japan). The following antibodies were used: anti-ABCC10/MRP7 (MyBiosource, San Diego, CA), anti-α-tubulin (Sigma Aldrich Biotechnology, St. Louis, MO) and HRP-conjugated secondary antibody (Cell Signaling Technology, Danvers, MA). α-tubulin was used as a loading control. The band intensities were analyzed by Image Quant TL (GE Healthcare Bioscience, Amersham Place, UK).

### Study population

The study included 69 patients with advanced NSCLC who were treated with docetaxel monotherapy (1-h intravenous infusion of 60 mg/m^2^) as a second cytotoxic chemotherapy (tyrosine kinase inhibitors were not counted as a cytotoxic chemotherapy) at Nagoya City University Hospital between January 2010 and December 2016. Written informed consent was obtained from all patients, and all routine medical data were anonymized. Approval for the study was obtained from the Ethics Committee of Nagoya City University. Other eligibility criteria included age (18 years or older), normal liver function, and Eastern Cooperative Oncology Group (ECOG) performance status (less than 2). Patients to whom granulocyte colony stimulating factor (G-CSF) was prophylactically administered were excluded. The dose reduction was made at the physician’s discretion based on the degree of adverse events.

### Genomic DNA extraction and detection of drug transporter polymorphisms

Genomic DNA was extracted from 18 NSCLC cell lines and blood samples from the 69 NSCLC patients using a QIAamp DNA Mini Kit (Qiagen) according to the manufacturer’s instructions. Drug transporter SNPs were detected using a StepOnePlus Real-Time PCR System (Applied Biosystems; Foster City, CA) and TaqMan SNP Genotyping Assays [*ABCC10* (rs9349256, C_1701942_10; rs2125739, C_16173668_10), *ABCB1* (C1236T, C_7586662_10; C3435T, C_7586657_20; G2677 T, C_11711720D_40; G2677A, C_11711720C_30), *ABCC2* (rs12762549, C_11214917_10), and *SLCO1B3* (rs11045585, C_31106434_10). All assays were purchased from Applied Biosystems and used in accordance with the manufacturer’s instructions. The risk alleles of these SNPs were selected by reference to previous reports [11, 12, 21].

### Statistical analysis

Differences between samples were evaluated by Mann Whitney U test. Efficacy was assessed by measurable disease based on the Response Evaluation Criteria in Solid Tumors (RECIST), version 1.0. All adverse events were graded using the Common Terminology Criteria for Adverse Events (CT-CAE), version 3.0. Survival curves of progression-free survival (PFS) and overall survival (OS) based on genotype were calculated using the Kaplan-Meier method and were compared using the log-rank test. The relationship between SNP genotype and response rate (RR) or adverse events was evaluated by Fisher’s exact test. The level of significance was set at 5% with two-sided analysis. Associations between drug transporter genotypes and the frequency of Grade 3/4 neutropenia for docetaxel were estimated using a forced entry logistic regression model with odds ratios (ORs) and 95% confidence intervals (CIs), adjusted for age and gender status; these variables mainly affect the degree of myelosuppression through pharmacokinetics in a clinical setting. All statistical analyses were performed with EZR (Saitama Medical Center, Jichi Medical University, Saitama, Japan), which is a graphical user interface for R (The R Foundation for Statistical Computing, Vienna, Austria). More precisely, it is a modified version of R commander designed to add statistical functions frequently used in biostatistics [22].

## Results

### Relationship between genetic variation in *ABCC10* and docetaxel cytotoxicity

**Fig. 1:**
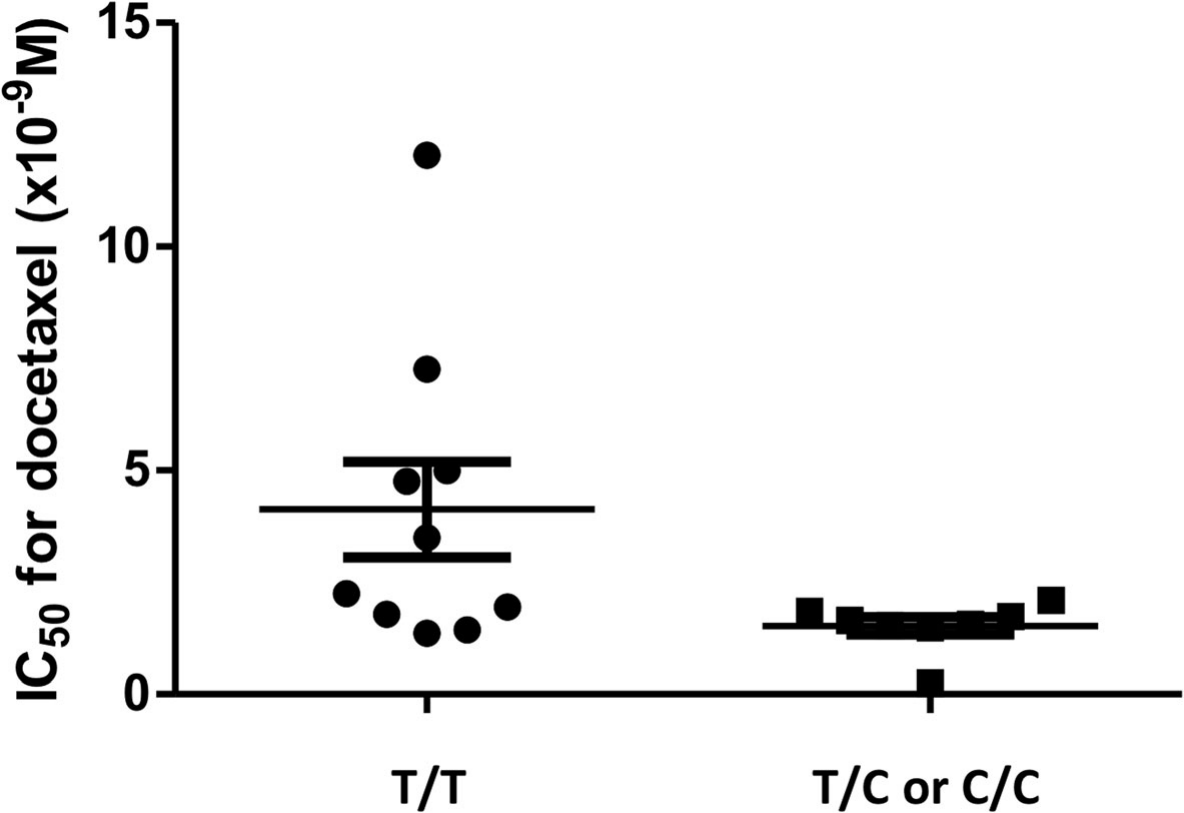
The relationship between the SNP rs2125739 genotype and in vitro docetaxel cytotoxicity. Ten cell lines carried the T/T variant, 4 carried the T/C variant, and 4 carried the C/C variant. The 50% inhibitory concentration (IC_50_) of the T/T group was significantly higher than that of the combined T/C and C/C group (*P* = 0.030). Each bar represents the mean ± SD within each variant group

Firstly, we examined the relationship between the *ABCC10* genotype and the in vitro cytotoxicity of docetaxel in 18 NSCLC cell lines. We found that the genetic variant rs2125739 was significantly associated with docetaxel cytotoxicity (10 cell lines carried the T/T variant, 4 carried the T/C variant, and 4 carried the C/C variant), with the T/T group showing a significantly higher IC_50_ compared to the combined T/C and C/C group; *P* = 0.030, Fig. 1). However, other genetic variations in *ABCC10* (rs9349256), *ABCB1* (C3435T, G2677 T/A and C1236T), *ABCC2* (rs12762549) or *SLCO1B3* (rs11045585) showed no significant correlation with docetaxel sensitivity. These SNPs were selected by reference to previous reports [11–13, 19, 21].

### Differences in docetaxel sensitivity between the T/T variant and T/C variant of rs2125739 in CRISPR-based genome edited cells

**Fig. 2:**
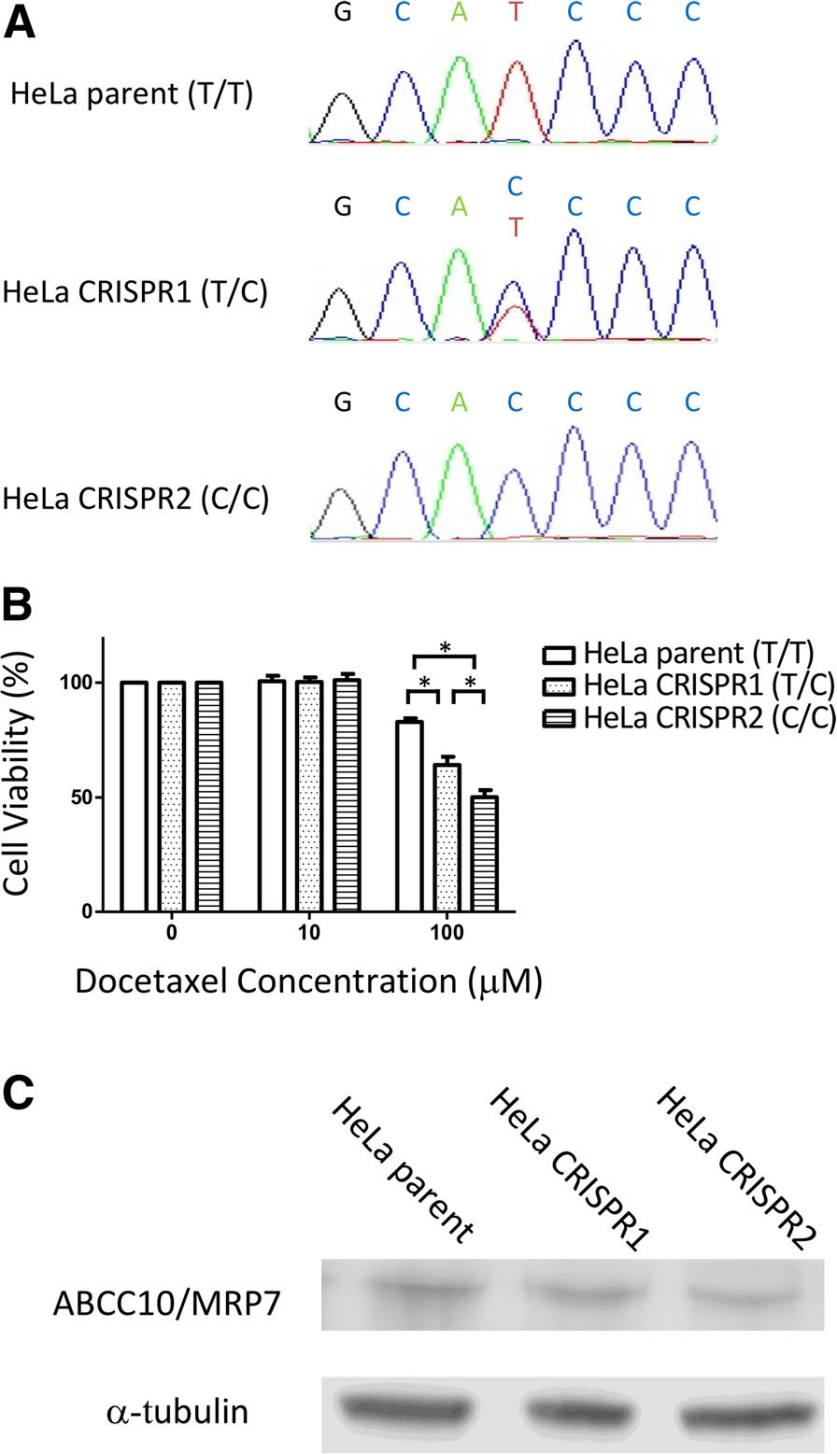
**a**. The Sangar sequencing results around rs2125739 of the genomic DNA samples from HeLa parent cells (T/T variant in rs2125739), HeLa CRISPR1 cells (T/C) and CRISPR2 cells (C/C). **b**. The differences in cytotoxicity to docetaxel between the parent and CRISPR-based genome-edited cells. HeLa CRISPR1 cells (T/C) and CRISPR2 cells (C/C) had significantly higher sensitivity to 100 μM docetaxel than did the HeLa parent cells (T/T). Similarly, HeLa parent cells (T/T) had a higher IC_50_ compared to HeLa CRISPR1 cells (T/C) and CRISPR2 cells (C/C) (T/T vs. T/C vs. C/C; 2.558 nM (95% CI = 2.051–3.192) vs. 1.246 nM (95% CI = 0.8581–1.810) vs. 0.8546 nM (95% CI = 0.6897–1.059), respectively). In this assay, 20 μM verapamil was added to the medium. Each bar represents the mean ± SD of three independent measurements. *P < 0.001. **c**. The protein expression of ABCC10/MRP7 was the same in HeLa parent cells (T/T), HeLa CRISPR1 cells (T/C) and CRISPR2 cells (C/C) as determined by western blotting

To confirm the relationship between the genetic variant rs2125739 and docetaxel sensitivity, we demonstrated the conversion of allele “T” to “C” through CRISPR-Cas9 genome-editing in HeLa cells (T/T variant in rs2125739) (Fig. 2a). The HeLa parent cells (T/T) showed a significantly higher sensitivity to docetaxel than the HeLa CRISPR1 cells (T/C) and CRISPR2 cells (C/C) (Fig. 2b). In this assay, 20 μM of verapamil, an inhibitor of ABCB1, was added to the medium because ABCB1 is moderately expressed in HeLa cells [23] and the inhibition of ABCB1 clarifies the difference in ABCC10 function in each genotype. ABCC10/MRP7 protein expression was the same in the HeLa parent cells and the HeLa CRISPR-edited cells (Fig. 2c). The position that is recognized by the monoclonal anti-ABCC10/MRP7 antibody (aa194–272) is far from the position of rs2125739 (Ile948Thr).

### Patient characteristics

The clinical characteristics of the 69 NSCLC patients are listed in Table 1. The cohort comprised 53 males and 16 females with a median age of 68 (range 41–79 years). Histological features were adenocarcinoma (*n* = 39), squamous-cell carcinoma (*n* = 25), and other non-small cell carcinoma (*n* = 5). Eleven patients had Stage IIIB disease and 58 patients had Stage IV. All patients were treated with a 1-h intravenous infusion of docetaxel (60 mg/m^2^) monotherapy as a second cytotoxic chemotherapy. Six patients showed a partial response (PR), 23 patients showed stable disease (SD), and 33 patients showed progressive disease (PD) (7 patients were not evaluable). The overall RR was 9.7% (6/62). The median PFS was 53 days, and the median OS was 284 days.

**Table 1:** Patient Characteristics (*n* = 69)

Characteristics	Value
Age (years)	
Median	68
Range	41–79
Sex, n	
Male	53
Female	16
Smoking status, n	
Current or Former smoker	59
Never smoked	10
Histological type, n	
Adenocarcinoma	39
squamous cell carcinoma	25
others	5
Disease stage, n	
IIIB	11
IV	58
Driver mutation status, n	
EGFR mutation	8
ALK translocation	1
negative or unknown	60
No. of prior systemic regimens, n	
1	61
> 2	8
Prior platinum drug therapy, n	
Yes	64
No	5
No. of docetaxel cycles	
Median	2
Range	1–14
Overall response for docetaxel	
Complete Response	0
Partial Response	6
Stable Disease	23
Progressive Disease	33
Not Evaluable	7
Neutropenia in first cycle of docetaxel	
Grade 0	3
Grade 1	5
Grade 2	5
Grade 3	17
Grade 4	39
Grade 5	0

*EGFR* epidermal growth facter receptor, *ALK* anaplastic lymphoma kinase

### Genetic variants of transporters and docetaxel treatment outcome

**Fig. 3:**
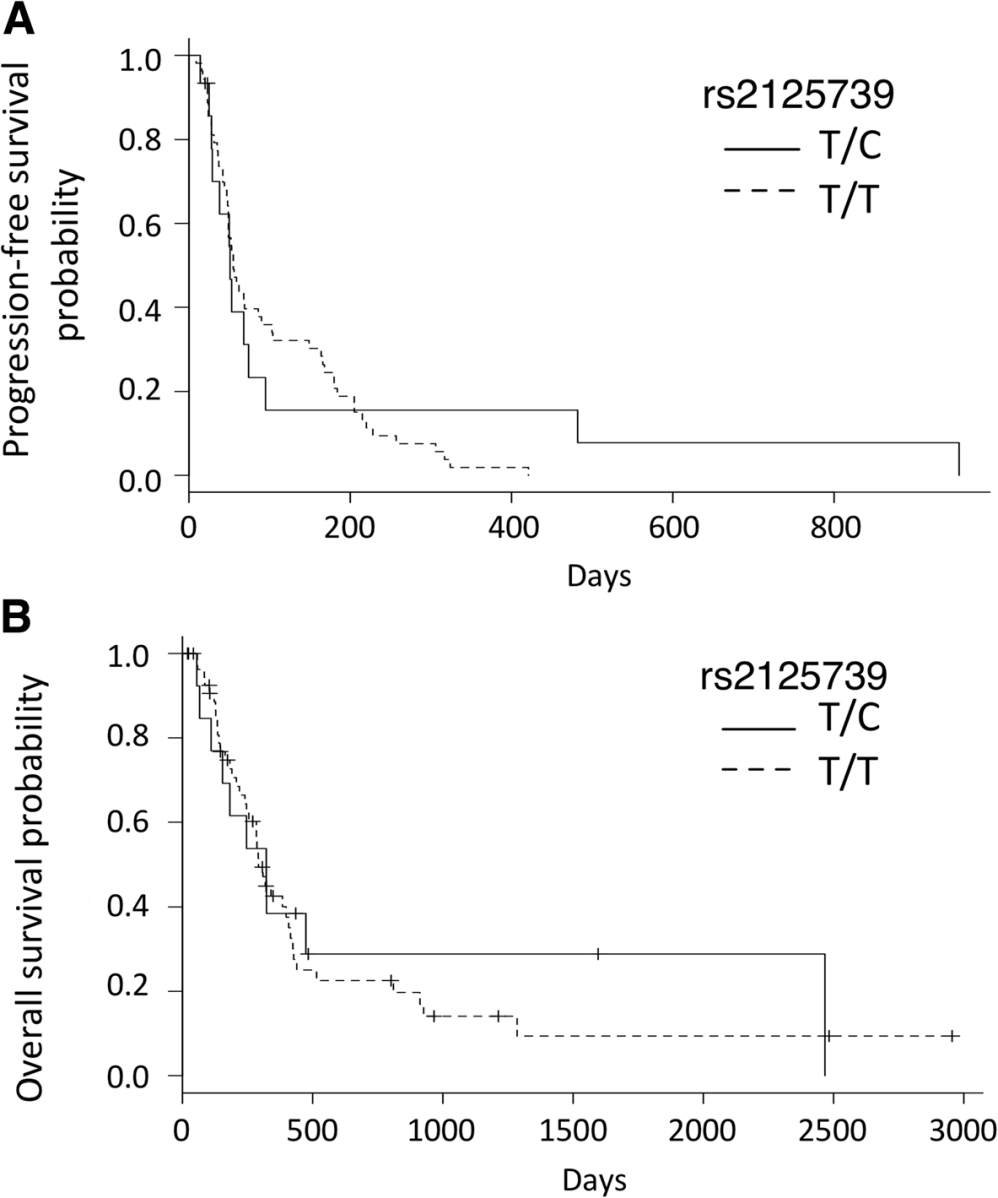
Kaplan–Meier survival curves of progression-free survival (PFS) (**a**) and overall survival (OS) (**b**) according to the SNP rs2125739 genotype. The median PFS in patients with the T/C or T/T variant was 51 days (95% confidence interval (CI), 28–74 days) and 55 days (95% CI, 48–90 days), respectively (*p* = 0.626). The median OS in patients with the T/C or T/T variant was 322 days (95% CI, 110-not available days) and 292 days (95% CI, 240–408 days), respectively (*p* = 0.748)

We examined the *ABCC10* SNP rs2125739 in 69 NSCLC patients in order to assess a potential relationship with clinical outcome. Fifteen patients carried the T/C variant, and 54 patients carried the T/T variant. However, there were no significant differences between the T/C and T/T groups in terms of RR (14.3% vs. 8.3%; *P* = 0.61), PFS (median PFS, 51 days vs. 55 days; *P* = 0.63), or OS (median OS, 322 days vs. 292 days; *P* = 0.75; Fig. 3). The alternative *ABCC10* variant (rs9349256) was not associated with RR, PFS, and OS (data not shown). In contrast however, the rs2125739 genotype was associated with grade 3/4 neutropenia after docetaxel treatment (T/C group 60% vs. T/T group 87%; *P* = 0.028; Table 2). In multivariate analysis, the OR for neutropenia adjusted by age and sex was significantly lower for those with the T/C variant of rs2125739, and there was no patient with febrile neutropenia in this group (T/C group 0% vs. T/T group 20.4%). SNP genotypes for the other transporters (*ABCB1*, *ABCC2*, and *SLCO1B3*) were not significantly associated with the frequency of neutropenia.

**Table 2:** Associations between the genetic polymorphism and neutropenia for docetaxel treatment in the 69 NSCLC patients

SNP	Genotype	n	Grade 3 or 4 neutropenia, %	*p*-value	febrile neutropenia, n	p-value	adjusted odds ratio^a^	*p*-value
*ABCC10* rs2125739	TT	54	87.0%	0.028	11	0.105		0.013
	TC	15	60.0%		0		0.16 (0.03–0.68)	
*ABCB1* 3435C > T	CC	25	88.0%	0.349	4	1.000		0.345
	CT + TT	44	77.3%		7		0.50 (0.12–2.09)	
*ABCB1* 2677G > T/A	GG	10	100.0%	0.189	3	0.192		–
	GT + TT + GA + AA+TA	59	78.0%		8		–	
*ABCB1* 1236C > T	CC	12	75.0%	0.685	0	0.191		0.546
	CT + TT	57	82.5%		11		1.60 (0.35–7.31)	
*ABCC2* rs12762549	CC	12	100.0%	0.104	1	0.674		–
	CG + GG	57	77.2%		10		–	
*SLCO1B3* rs11045585	GG	54	77.8%	0.270	9	1.000		0.185
	GA	15	93.3%		2		4.31 (0.50–37.3)	

^a^odds ratio of frequency of grade 3/4 neutropenia adjusted by age and sex in a logistic regression analysis

SNP, single nucleotide polymorphism

## Discussion

In this study, we found that genetic variation within the *ABCC10* gene is associated with docetaxel cytotoxicity in NSCLC cell lines and CRISPR edited cells, and with neutropenia in NSCLC patients treated with this drug. We believe this to be the first report describing a relationship between the *ABCC10* genotype and the clinical outcome of patients to docetaxel treatment.

ABC transporters are important in drug uptake, distribution, and elimination in normal tissues, resulting in the protection of cells from many toxic insults [8]. However, the cellular protection afforded by ABC transporter-mediated extrusion also extends to chemotherapeutic drugs. Recent studies have indicated that *ABCC10* knockout mice have no obvious health problems, indicating that the gene is not essential for normal physiological functions [24]. In contrast, *ABCC10* knockout mice treated with paclitaxel exhibited increased lethality associated with neutropenia and marked bone marrow toxicity. These results indicate that the genetic deficiency in *ABCC10* results in increased tissue sensitivity to taxanes compared with wild-type mice, arguing that ABCC10/MRP7 functions as a major determinant of sensitivity to taxane compounds. The exonic SNP in *ABCC10* (rs2125739) was associated with docetaxel sensitivity in the present study. A possible mechanism of this is that rs2125739, as is located in a putative splicing site, affects pre-mRNA splicing and leads to an altered protein [25]. On the other hand, each protein expression levels of ABCC10/MRP7 was the same in parent cells and CRISPR edited cells in the present study. Therefore, the functional ability of ABCC10/MRP7 to transport docetaxel may be affected by this genetic variant through not expression level but structural differences in the ABCC10/MRP7 protein.

The main dose-limiting toxicity of docetaxel is neutropenia [5, 6]. A previous phase 3 trial reported that grade 3/4 neutropenia occurred in 73.6% of Japanese NSCLC patients treated with docetaxel as a second-line therapy [26]. In our retrospective study, grade 3/4 neutropenia was found in 81.2% of patients, a similar incidence as in this previous report. Docetaxel pharmacokinetics has been shown to be predictive of hematologic toxicity, especially in both grade 4 neutropenia and febrile neutropenia [27], and the elimination of docetaxel is primarily via the biliary and hepatic routes [6]. Because ABCC10/MRP7 is highly expressed in the liver [17], ABCC10/MRP7 may also play a role in the metabolism of docetaxel in this organ. We found that genetic variation in *ABCC10* (rs2125739) was associated with the cytotoxicity of docetaxel in 18 lung cancer cell lines and this was confirmed by CRISPR-based genome editing, suggesting that the *ABCC10* genotype may influence the ABCC10/MRP7 ability to deliver docetaxel and the cytoplasmic drug concentration of docetaxel. Considering this, the *ABCC10* genotype may affect the plasma concentration of docetaxel. This hypothesis is consistent with the observation that the rs2125739 polymorphism is significantly associated with the plasma concentration of nevirapine, a non-nucleoside reverse transcriptase inhibitor for HIV-1 infection that is also a substrate for ABCC10/MRP7 [18]. Moreover, it has been previously reported that the knockout of drug transporters has a significant impact on docetaxel clearance [28]. Although we did not examine the pharmacokinetics of docetaxel in this study, these data suggest that genetic variation in *ABCC10* may influence the pharmacokinetics of docetaxel, resulting in the observed relationship with grade 3/4 neutropenia and/or neutropenic fever. In contrast, and unlike the findings from past studies, the SNP genotypes of other transporters were not associated with docetaxel neutropenia in this study. This may be related to the small number of patients used in our study and/or to cohort differences (i.e., a higher frequency of grade 3/4 neutropenia compared to previous studies). However, the finding suggests that *ABCC10* may play a more important role than other transporters, since this was the only gene which was associated with both in vitro docetaxel cytotoxicity and clinical neutropenia in this study.

In previous studies, ABCC10/MRP7 has been shown to mediate the ATP-dependent transport of taxanes, conferring taxane resistance to cancer cells [9, 14]. In our retrospective study, the RR was 9.7% and the PFS was 53 days. These data are similar to other results from Japanese NSCLC patients treated with docetaxel as second-line therapy, in which the RR was 12.8% and the PFS was 2 months [5]. We found an association between the *ABCC10* genotype and docetaxel cytotoxicity in cell lines but did not find any significant relationship with the clinical efficacy of docetaxel treatment of NSCLC patients. One of the reasons for this discrepancy is considered to be the existence of other drug resistance mechanisms. Although drug efflux by ABC transporters appears to be an important mechanism of resistance to taxanes, alterations in microtubule structure, resulting in altered microtubule dynamics and/or altered binding of taxanes, may also be a significant determinant of the activity of docetaxel [29]. The critical mechanism of action of these drugs, as it relates to clinical efficacy, thus remains to be determined. In contrast to our findings, genetic variability in *ABCC10* (rs2125739) has been associated with survival outcome in colorectal cancer patients receiving oxaliplatin-based chemotherapy [19]. Considering that the population in the present study represented previously treated patients and that prior chemotherapy may affect *ABCC10* expression, it may be that the rs2125739 variant is related to the efficacy of docetaxel-based chemotherapy only in the front-line setting.

The major limitation of the present study is that it is based on a retrospective analysis of a small number of patients with advanced NSCLC in a single institute, and there may thus be potential bias with regard to patient selection and follow-up. Furthermore, genetic variants generally have ethnic differences; indeed, variation in rs2125739 has been associated with nevirapine plasma concentration primarily in Caucasian patients [18]. Further validation in other cohorts or other carcinomas is thus needed to corroborate the present results. In addition, this study did not conduct exhaustive analysis of the effector gene of rs2125739, and there is a possibility that the SNP rs2125739 regulates genes other than *ABCC10*. However, we consider that rs2125739 mainly contributes to the ABCC10/MRP7 function of docetaxel delivery because rs2125739 is a non-synonymous SNP in the *ABCC10* gene.

The clinical development of docetaxel is progressing even in recent years. The clinical trial in previously treated patients with NSCLC showed that docetaxel combined with the angiogenesis inhibitor ramcirumab improved survival, however, the incidence of neutropenia and febrile neutropenia in patients treated with the combination therapy was higher than with docetaxel alone [30]. As docetaxel is still an important cytotoxic agent and new therapies for various cancers (such as immunotherapy) are coming up, the prediction of neutropenia that is a dose-limiting toxicity for docetaxel is critical to select treatment. Moreover, this genetic variation may be a reference for the adaptation of prophylactical G-CSF. Several genome-wide associated studies for taxanes, including docetaxel, have been performed but the results are inconsistent [31–33]. Therefore, the approach from the physiological mechanism of drug metabolism is also important. Further prospective studies of the relationship between the *ABCC10* genotype and the incidence of neutropenia and/or neutropenic fever following docetaxel treatment are now warranted.

## Conclusion

Our results indicate that genetic variation in the *ABCC10* gene is associated with neutropenia for docetaxel treatment.

## Abbreviations

ABC: ATP binding cassette
CI: Confidence interval
CT-CAE: Common Terminology Criteria for Adverse Events
ECOG: Eastern Cooperative Oncology Group
G-CSF: Granulocyte colony stimulating factor
IC_50_: Drug concentration resulting in 50% growth inhibition
MRP7: Multidrug resistance protein 7
NSCLC: Non-small lung cancer
ORs: Odds ratios.
OS: Overall survival
PD: Progressive disease
PFS: Progression free survival
PR: Partial response
RECIST: Response Evaluation Criteria in Solid Tumors
RR: Response rate
SD: Stable disease
SNP: Single nucleotide polymorphism
